# Comparative Label-Free Mass Spectrometric Analysis of Mildly *versus* Severely Affected *mdx* Mouse Skeletal Muscles Identifies Annexin, Lamin, and Vimentin as Universal Dystrophic Markers

**DOI:** 10.3390/molecules200611317

**Published:** 2015-06-19

**Authors:** Ashling Holland, Michael Henry, Paula Meleady, Claudia K. Winkler, Mirjam Krautwald, Heinrich Brinkmeier, Kay Ohlendieck

**Affiliations:** 1 Department of Biology, Maynooth University, Maynooth, Co. Kildare, Ireland; E-Mail: ashling_holland@hotmail.com; 2 National Institute for Cellular Biotechnology, Dublin City University, Dublin 9, Ireland; E-Mails: michael.henry@dcu.ie (M.H.); paula.meleady@dcu.ie (P.M.); 3 Institute of Pathophysiology, University Medicine Greifswald, D-17495 Karlsburg, Germany; E-Mails: winklerc@uni-greifswald.de (C.K.W.); mirjam.krautwald@uni-greifswald.de (M.K.); heinrich.brinkmeier@uni-greifswald.de (H.B.)

**Keywords:** diaphragm, dystrophin, dystrophinopathy, Duchenne muscular dystrophy, *extensor digitorum longus*, *flexor digitorum brevis*, *interosseus*, muscle pathology, *soleus*, skeletal muscle proteome

## Abstract

The primary deficiency in the membrane cytoskeletal protein dystrophin results in complex changes in dystrophic muscles. In order to compare the degree of secondary alterations in differently affected subtypes of skeletal muscles, we have conducted a global analysis of proteome-wide changes in various dystrophin-deficient muscles. In contrast to the highly degenerative *mdx* diaphragm muscle, which showed considerable alterations in 35 distinct proteins, the spectrum of mildly to moderately dystrophic skeletal muscles, including *interosseus*, *flexor digitorum brevis*, *soleus*, and *extensor digitorum longus* muscle, exhibited a smaller number of changed proteins. Compensatory mechanisms and/or cellular variances may be responsible for differing secondary changes in individual *mdx* muscles. Label-free mass spectrometry established altered expression levels for diaphragm proteins associated with contraction, energy metabolism, the cytoskeleton, the extracellular matrix and the cellular stress response. Comparative immunoblotting verified the differences in the degree of secondary changes in dystrophin-deficient muscles and showed that the up-regulation of molecular chaperones, the compensatory increase in proteins of the intermediate filaments, the fibrosis-related increase in collagen levels and the pathophysiological decrease in calcium binding proteins is more pronounced in *mdx* diaphragm as compared to the less severely affected *mdx* leg muscles. Annexin, lamin, and vimentin were identified as universal dystrophic markers.

## 1. Introduction

The full-length isoform of the membrane cytoskeletal actin-binding protein dystrophin, termed Dp427, represents the product of the largest gene in the human genome [1,2,3]. Although the Dp427 isoform is not an integral protein, it is tightly associated with the muscle sarcolemma via linkage to a large membrane-embedded assembly of glycoproteins [4,5,6]. The key trans-plasmalemma spanning protein within this dystrophin-associated complex was shown to be β-dystroglycan, a glycosylated protein of 43 kDa, which in turn binds to the extracellular laminin-receptor named α-dystroglycan of 156 kDa [7]. Since β-dystroglycan anchors dystrophin of 427 kDa to the surface membrane, the dystrophin-glycoprotein complex provides a connection between the actin membrane cytoskeleton of the fibre interior and the laminin network of the basal lamina and extended extracellular matrix that surrounds contractile cells [8]. The dystrophin lattice of the sarcolemma, in combination with the dystrophin-associated protein complex, is believed to stabilize muscle fibres during the repetitive physical strains of continuous excitation-contraction-relaxation cycles, and also functions as a binding partner for signalling molecules and ion channels [9,10]. It is therefore not surprising that the loss of function of such a crucial stabilizing component of the muscle fibre periphery causes severe secondary alterations in affected skeletal muscle fibres [6].

The disease grouping of dystrophinopathies is based on mutations or genetic rearrangements in the 79-exon spanning dystrophin gene [3]. Disorders of the skeletal musculature and the heart include highly progressive Duchenne muscular dystrophy, which is an early-onset and debilitating dystrophinopathy, Becker muscular dystrophy, which is a delayed-onset and milder dystrophinopathy, and X-linked dilated cardiomyopathy, which afflicts teenage men [11,12,13]. In muscular dystrophy, the absence of dystrophin isoform Dp427 was shown to cause a variety of physiological and biochemical changes, including a higher susceptibility to stretch-induced injury, impaired excitation-contraction coupling, lowered luminal calcium buffering, an elevated sarcolemmal influx of calcium ions and a concomitant increase in the proteolytic degradation of muscle proteins [14,15,16].

Model organisms have been instrumental in muscular dystrophy research, such as zebrafish, the *mdx* mouse and the *grmd* dog, including biomarker discovery studies [17,18,19,20]. Although genocopies of an inherited disorder often do not show the same clinical phenotype in animal models due to differences in down-stream pathophysiological changes, the *mdx* mouse model of Duchenne muscular dystrophy is highly suitable for biomedical studies and the initial evaluation of novel treatment strategies. Since the same primary abnormality, *i.e.*, a single base substitution in exon 23 of the dystrophin gene that causes the premature termination of the Dp427 polypeptide chain, results in a great variety of changes in different subtypes of muscle tissue, the *mdx* mouse is an ideal system to test novel drugs or experimental treatments, such as myoblast transfer approaches or exon-skipping therapy [19]. While extraocular, laryngeal, and *interosseus* muscle only exhibit marginal changes, the limb musculature undergoes segmental necrosis and the diaphragm muscle shows severe fibre wasting and fibrosis in the *mdx* mouse. These differing degrees of histopathological changes are clearly reflected by the extent of proteome-wide alterations in dystrophic muscles [21,22,23,24,25].

Since these striking variations in the degree of muscle degeneration in the differing dystrophic phenotypes within the same organism must be due to dissimilar secondary effects and/or compensatory mechanisms, proteomics suggests itself as an ideal analytical tool to determine global differences between the various muscle subtypes in dystrophic animal models. Building on the findings from previous proteomic surveys of muscular dystrophy, as recently outlined in comprehensive reviews [26,27,28], we have determined here differential protein expression patterns in mildly *vs.* severely affected dystrophic *mdx* mouse skeletal muscles. The comparative label-free mass spectrometric analysis of the severely dystrophic and fibrotic diaphragm, the relatively mildly affected *interosseus* and *flexor digitorum brevis* muscles, and the moderately dystrophic *soleus* and *extensor digitorum longus* muscles identified annexin, lamin, and vimentin as universal dystrophic markers.

## 2. Results and Discussion

### 2.1. Comparative Label-Free Mass Spectrometric Analysis of mdx Skeletal Muscles

**Figure 1:**
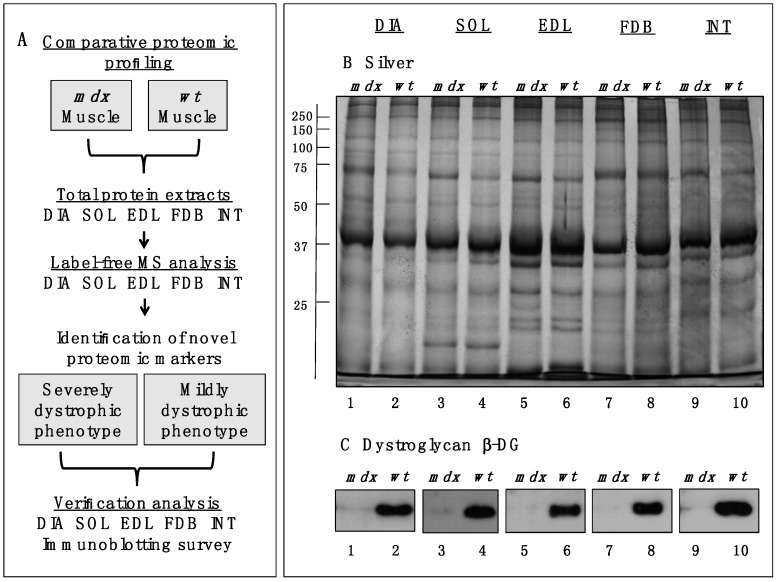
Comparative profiling of wild type (*wt*) muscles *vs.* dystrophic diaphragm (DIA), *soleus* (SOL), *extensor digitorum longus* (EDL), *flexor digitorum brevis* (FDB), and *interosseus* (INT) muscles from the *mdx* animal model of Duchenne muscular dystrophy. (**A**) Workflow of the comparative proteomic analysis of normal *vs.* dystrophic skeletal muscles; (**B**) Silver-stained gel of the *mdx* and *wt* preparations from DIA (lanes 1 and 2), SOL (lanes 3 and 4), EDL (lanes 5 and 6), FDB (lanes 7 and 8), and INT (lanes 9 and 10) muscles; (**C**) Comparative immunoblot analysis of β-dystroglycan showing antibody labelling of this dystrophin-associated glycoprotein of 43 kDa in normal *vs.* dystrophic muscles.

**Figure 2:**
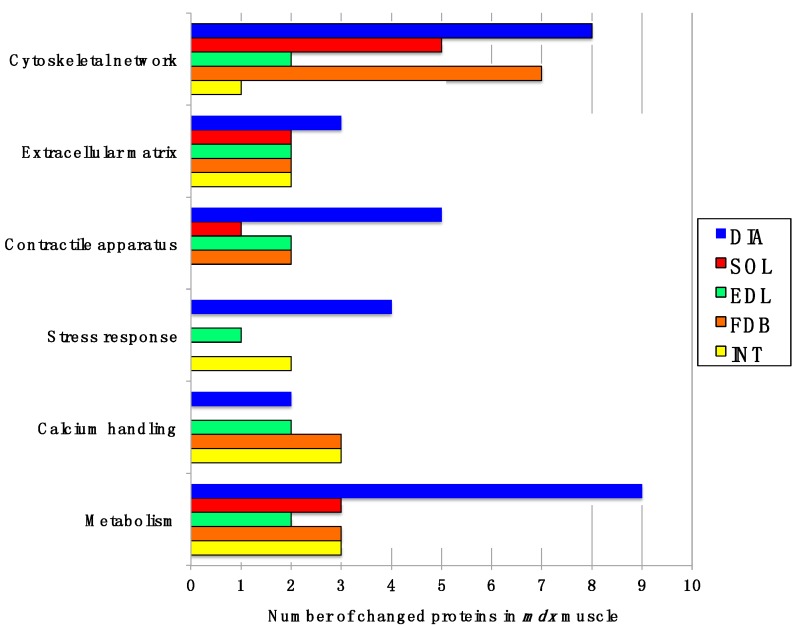
Diagrammatic presentation of the number of changed proteins in dystrophic diaphragm (DIA), *soleus* (SOL), *extensor digitorum longus* (EDL), *flexor digitorum brevis* (FDB), and *interosseus* (INT) muscles from the *mdx* animal model of Duchenne muscular dystrophy. The graph outlines affected functional protein families associated with the cytoskeleton, the extracellular matrix, the contractile apparatus, the cellular stress response, Ca^2+^-handling and metabolism.

In order to identify common proteomic markers that exhibit a changed concentration in differently affected *mdx* skeletal muscles, total extracts from diaphragm (DIA), *soleus* (SOL), *extensor digitorum longus* (EDL), *flexor digitorum brevis* (FDB), and *interosseus* (INT) muscles were analysed by label-free mass spectrometry. Figure 1A,B outlines the workflow of this analytical strategy and shows a representative silver-stained gel of the various preparations used in this study. The immunoblot analysis of β-dystroglycan demonstrated a drastic reduction of this dystrophin-associated glycoprotein of 43 kDa in all *mdx* muscles investigated (Figure 1C) [29,30]. The below displayed Table 1 to Table 5 list the results for each analysed skeletal muscle detailing the accession numbers of identified proteins, the number of peptides used in the analysis, the MS scores, ANOVA values and the fold change. The comparative study revealed an altered abundance in 35, 16, 18, 23 and 14 proteins in DIA, SOL, EDL, FDB and INT preparations, respectively. The total number of positively identified proteins in wild type DIA, SOL, EDL, FDB, and INT was 296 ± 17, 270 ± 13, 202 ± 11, 207 ± 7 and 215 ± 4, respectively, and in *mdx* DIA, SOL, EDL, FDB and INT was 348 ± 20, 289 ± 4, 218 ± 6, 222 ± 11 and 235 ± 16, respectively. The comparison of the differences in the number of changes between individual subtypes of muscles and total numbers of identified proteins suggests, therefore, that the observed effects are mostly based on pathobiochemical variations and not technical issues associated with sample preparation. The direct comparison of proteomic findings related to the most severely affected DIA muscle *vs.* mildly dystrophic INT muscle illustrates this conclusion. Although 1.4-fold more protein species were overall identified in DIA preparations, the rate of changes in DIA muscle is 2.5 fold higher as compared to INT preparations (Table 1 and Table 5). This indicates that the variation in the number of changes between individual muscle subtypes is due to biological effects rather than technical matters. Figure 2 gives an overview of the number of changed proteins in individual *mdx* muscles and their association with distinct functional families, such as the cytoskeleton, the extracellular matrix, the contractile apparatus, the cellular stress response, Ca^2+^-homeostasis and metabolism. As can be deduced from Figure 2 and Table 1, the *mdx* DIA muscle showed both quantitatively and qualitatively the largest secondary changes due to deficiency in dystrophin. Changed expression levels were shown for proteins mostly involved in fibre contraction, energy metabolism, metabolite transportation, the cytoskeleton, the extracellular matrix and the cellular stress response.

### 2.2. Proteomic Analysis of mdx Diaphragm Muscle

As listed in Table 1, 28 proteins exhibited an increased concentration and 7 proteins a decreased abundance in dystrophic DIA muscle preparations. Overall, our analysis identified 103 changed DIA proteins including those that were recognized only by one peptide (not shown). The most drastically elevated expression levels with values of 2-fold or higher were established for myosin-8, myosin-3, protein disulphide isomerase, lamin-A/C, myosin light chain 6B, vimentin, obscurin, annexin A5, hemopexin, the collagen alpha-1(VI) and alpha-2(VI) chains, lamin-B1, histone H3.2, moesin, albumin, tubulin, and the 78 kDa glucose-regulated protein. Myosin heavy chain isoforms myosin-3 and myosin-8 are embryonic isoforms and important markers of skeletal muscle regeneration. Their substantially increased concentration suggests both fibre regeneration and the compensatory re-organization of myofibrils within dystrophic fibres. The perinatal myosin-8 isoform has been identified in mature skeletal muscles by shotgun proteomics [31] and its elevated levels in the *mdx* diaphragm suggests the potential recruitment of new myofibre populations containing embryonic isoforms of myosin heavy chains. Importantly, high levels of the intermediate filament protein vimentin in *mdx* diaphragm muscle were also previously described to occur in EDL, FDB, GAS (*gastrocnemius*), INT, SOL, and VL (*vastus lateralis*) muscles from various dystrophin-deficient mouse and dog models of X-linked muscular dystrophy [21,23,24,25,32,33,34,35,36]. This establishes this protein as an interesting biomarker candidate that might be extremely helpful to evaluate animal models of dystrophinopathy. Changes in annexins, such as isoforms A1, A2, A5, or A6, are also an established alteration in dystrophic muscle tissues [25,33,37,38]. The specific increase in annexin A5 in the 100-day old *mdx* diaphragm agrees with the proteomic profiling of the senescent *mdx* diaphragm muscle by two-dimensional fluorescence difference in-gel electrophoresis [37] and the six-month old *mdx* hind limb muscles using standard two-dimensional gel electrophoresis combined with silver staining [38].

Moderately increased levels, ranging from approximately 1.5- to 2-fold change, were shown for transferrin, filamin-C, histone H1.2, elongation factor EEF2, the heat shock proteins Hsp90-beta, Hsp71 cognate and Hsp beta-1, the SERCA2 isoform of the sarcoplasmic reticulum Ca^2+^-ATPase, desmin, vinculin, and anti-trypsin. Decreased proteins were identified as the muscle-specific isoform of glycogen phosphorylase, mitochondrial 3-ketoacyl-CoA thiolase, medium-chain specific acyl-CoA dehydrogenase, myozenin, myoglobin, parvalbumin, and carbonic anhydrase isoform CA3. In analogy to this study, a variety of molecular chaperones belonging to the Hsp70 and Hsp90 families of heat shock proteins [27] were also identified in gel-based studies of dystrophic muscles [23,37,39].

**Table 1:** List of changed proteins in 100-day old *mdx* diaphragm muscle *vs.* age-matched wild type muscle as determined by label-free mass spectrometric analysis.

Accession Number	Protein Name	Tissue Localization	Peptides	Score	ANOVA (p)	Fold Change
P13542	Myosin-8 (perinatal MHC)	Myofibrils (myosin complex)	3	518.76	1.88 × 10^−5^	798.68
P13541	Myosin-3 (embryonic MHC-3)	Myofibrils (myosin complex)	2	345.71	1.13 × 10^−5^	84.52
P09103	Protein disulfide-isomerase	Endoplasmic reticulum	2	154.39	1.39 × 10^−5^	3.35
P48678	Prelamin-A/C	Nuclear lamina	8	511.85	2.87 × 10^−7^	3.31
Q8CI43	Myosin light chain 6B	Myofibrils (myosin complex)	3	142.05	1.16 × 10^−5^	3.31
P20152	Vimentin	Intermediate filaments	5	434.45	1.90 × 10^−5^	3.10
A2AAJ9	Obscurin	Contractile apparatus	2	145.90	5.47 × 10^−5^	2.97
P48036	Annexin A5	Sarcolemma region	2	137.12	4.69 × 10^−4^	2.67
Q91X72	Hemopexin	Extracellular space	2	94.70	1.03 × 10^−4^	2.62
Q02788	Collagen alpha-2(VI) chain	Extracellular matrix	5	266.91	5.40 × 10^−4^	2.31
P14733	Lamin-B1	Nuclear envelope	2	127.83	5.32 × 10^−5^	2.23
Q6LBE8	Histone H3.2	Nucleus	2	149.87	1.23 × 10^−3^	2.20
P26041	Moesin	Cytoskeleton/sarcolemma	2	113.60	4.09 × 10^−4^	2.18
P07724	Serum albumin	Extracellular space	3	176.70	3.07 × 10^−3^	2.15
Q04857	Collagen alpha-1(VI) chain	Extracellular matrix	5	305.12	4.35 × 10^−4^	2.12
Q7TMM9	Tubulin beta-2A chain	Microtubules	3	188.58	2.56 × 10^−4^	2.05
P20029	78 kDa glucose-regulated protein	Endoplasmic reticulum	3	145.99	4.79 × 10^−4^	2.00
Q921I1	Serotransferrin	Extracellular space	5	257.43	6.48 × 10^−5^	1.99
Q8VHX6	Filamin-C	Actin cytoskeleton	16	1111.06	5.07 × 10^−7^	1.93
P15864	Histone H1.2	Nucleus	2	128.46	8.47 × 10^−3^	1.88
P58252	Elongation factor 2	Cytoplasm/ribosome	5	286.21	2.42 × 10^−5^	1.87
P11499	Heat shock protein Hsp90-beta (HSPAB1)	Cytoplasm	5	285.81	6.42 × 10^−5^	1.86
P63017	Heat shock cognate 71 kDa protein (HSPA8, Hsc70)	Cytoplasm	4	294.22	5.69 × 10^−4^	1.77
P14602	Heat shock protein beta-1 (HSPB1, Hsp27)	Cytoplasm	2	114.96	2.04 × 10^−4^	1.77
O55143	SERCA2 Ca^2+^-ATPase	Sarcoplasmic reticulum	2	106.96	3.43 × 10^−2^	1.72
P31001	Desmin	Intermediate filaments	6	478.06	1.41 × 10^−4^	1.71
Q64727	Vinculin	Cytoskeleton	2	145.57	7.51 × 10^−4^	1.68
Q00897	Alpha-1-antitrypsin 1-4	Extracellular region	3	148.95	1.47 × 10^−6^	1.63
Q9WUB3	Glycogen phosphorylase, muscle	Cytoplasm	7	418.32	1.10 × 10^−3^	−1.51
Q8BWT1	3-ketoacyl-CoA thiolase, mitochondrial	Mitochondrion	2	107.43	6.21 × 10^−3^	−1.55
P45952	Medium-chain specific acyl-CoA dehydrogenase	Mitochondrial matrix	2	112.72	4.60 × 10^−4^	−1.56
Q9JK37	Myozenin-1	Actin cytoskeleton	2	96.43	1.24 × 10^−3^	−1.97
P04247	Myoglobin	Cytoplasm	3	144.52	3.35 × 10^−4^	−2.73
P32848	Parvalbumin alpha	Cytoplasm	4	255.00	6.93 × 10^−4^	−2.83
P16015	Carbonic anhydrase CA3	Cytoplasm	3	192.85	3.38 × 10^−4^	−3.76

### 2.3. Proteomic Analysis of mdx Soleus Muscle

In the dystrophic SOL muscle, label-free mass spectrometric analysis revealed an increase of 2-fold or higher for vimentin, serpin B6, hemopexin, actin, annexin A2, and albumin (Table 2). A moderate elevation in expression levels was shown for lamin-A/C, elongation factors EEF1A1 and EEF2, antitrypsin, heat shock protein Hsp90-beta and the transitional endoplasmic reticulum ATPase. In contrast, dystrophin deficiency in SOL muscles was associated with decreases in lactate dehydrogenase, fatty acid-binding protein FABP3, myoglobin, and parvalbumin.

**Table 2:** List of changed proteins in 100-day old *mdx* *soleus* muscle *vs.* age-matched wild type muscle as determined by label-free mass spectrometric analysis.

Accession Number	Protein Name	Tissue Localization	Peptides	Score	ANOVA (p)	Fold Change
P20152	Vimentin	Intermediate filaments	5	462.30	3.75 × 10^−5^	5.44
Q60854	Serpin B6	Cytoplasm	2	136.46	8.01 × 10^−5^	3.34
Q91X72	Hemopexin	Extracellular space	2	98.63	3.10 × 10^−3^	2.63
P60710	Actin, cytoplasmic 1	Cytoplasm	2	86.84	6.69 × 10^−6^	2.47
P07356	Annexin A2	Sarcolemma/basal lamina	3	184.47	8.10 × 10^−5^	2.41
P07724	Serum albumin	Extracellular space	4	193.77	9.49 × 10^−3^	2.38
P48678	Prelamin-A/C	Nuclear lamina	5	313.84	4.15 × 10^−4^	1.84
P10126	Elongation factor 1-alpha 1	Cytoplasm/ribosome	4	207.63	1.05 × 10^−3^	1.82
Q00896	Alpha-1-antitrypsin 1-3	Extracellular region	2	109.93	1.33 × 10^−3^	1.71
P58252	Elongation factor 2	Cytoplasm/ribosome	4	242.28	1.50 × 10^−4^	1.63
P11499	Heat shock protein HSP 90-beta (HSPAB1)	Cytoplasm	6	373.77	1.98 × 10^−3^	1.61
Q01853	Transitional endoplasmic reticulum ATPase	Endoplasmic reticulum	2	104.50	5.72 × 10^−3^	1.59
P16125	l-lactate dehydrogenase B chain	Cytoplasm	2	129.92	8.59 × 10^−4^	−1.71
P11404	Fatty acid-binding protein FABP3	Cytoplasm	2	83.44	8.28 × 10^−4^	−1.84
P04247	Myoglobin	Cytoplasm	2	117.20	7.47 × 10^−4^	−2.10
P32848	Parvalbumin, alpha	Cytoplasm	3	186.18	1.02 × 10^−2^	−2.18

### 2.4. Proteomic Analysis of mdx Extensor Digitorum Longus Muscle

In the dystrophin-deficient EDL muscle, label-free mass spectrometric analysis showed a drastic increase of two-fold or higher for vimentin, albumin, annexin A2, lamin-A/C, serotransferrin, apolipoprotein A-I, histone H4, antitrypsin, heat shock proteins Hsp71 cognate, and Hsp90-beta. Moderate elevations were shown for the elongation factors EEF1A1 and EEF2, and phosphofructokinase (Table 3). The mass spectrometric analysis revealed decreases in myozenin-1, cytoplasmic aspartate aminotransferase, the alpha-1 and alpha-2 chains of collagen I, and myoglobin.

**Table 3:** List of changed proteins in 100-day old *mdx* *extensor digitorum longus* muscle *vs.* age-matched wild type muscle as determined by label-free mass spectrometric analysis.

Accession Number	Protein Name	Tissue Localization	Peptides	Score	ANOVA (p)	Fold Change
P20152	Vimentin	Intermediate filaments	8	561.29	4.53 × 10^−4^	7.32
P07724	Serum albumin	Extracellular space	5	250.52	1.80 × 10^−3^	4.02
P07356	Annexin A2	Sarcolemma/basal lamina	2	124.10	1.77 × 10^−4^	3.93
P48678	Prelamin-A/C	Nuclear lamina	4	267.71	7.35 × 10^−6^	3.50
Q921I1	Serotransferrin	Extracellular space	2	89.28	2.42 × 10^−2^	2.81
Q00623	Apolipoprotein A-I	Cytoplasm/extracellular space	3	234.45	1.82 × 10^−2^	2.55
P62806	Histone H4	Nucleus	2	123.52	2.26 × 10^−3^	2.41
Q00896	Alpha-1-antitrypsin 1-3	Extracellular region	2	93.70	3.09 × 10^−2^	2.35
P63017	Heat shock cognate 71 kDa protein (HSPA8, Hsc70)	Cytoplasm	5	289.78	3.56 × 10^−3^	2.04
P11499	Heat shock protein Hsp90-beta (HSPAB1)	Cytoplasm	2	105.43	5.75 × 10^−5^	2.02
P58252	Elongation factor 2	Cytoplasm/ribosome	3	185.26	1.63 × 10^−2^	1.85
P10126	Elongation factor 1-alpha 1	Cytoplasm/ribosome	2	91.27	4.50 × 10^−3^	1.53
P47857	6-phosphofructokinase, muscle	Cytoplasm/glycolytic particle	3	232.53	3.77 × 10^−3^	1.53
Q9JK37	Myozenin-1	Actin cytoskeleton	2	96.90	3.41 × 10^−2^	−1.75
P05201	Aspartate aminotransferase	Cytoplasm	2	123.54	2.64 × 10^−2^	−1.92
Q01149	Collagen alpha-2(I) chain	Extracellular matrix	3	142.26	1.99 × 10^−2^	−2.62
P11087	Collagen alpha-1(I) chain	Extracellular matrix	2	108.38	4.00 × 10^−2^	−2.91
P04247	Myoglobin	Cytoplasm	2	146.49	1.45 × 10^−3^	−3.43

### 2.5. Proteomic Analysis of mdx Flexor Digitorum Brevis Muscle

In the FDB muscle from the *mdx* mouse, label-free mass spectrometric analysis revealed increases in vimentin, annexin A2 and A5, cytoplasmic actin, apolipoprotein A-I, alpha-1-antitrypsin 1-2, albumin, serotransferrin, heat shock protein Hsp90-beta, lamin-A/C, lumican, protein disulfide-isomerase, and the alpha-1 and alpha-2 chains of collagen VI (Table 4). A decreased abundance was observed for the glycolytic enzyme triosephosphate isomerase, protein NDRG2, glycogen phosphorylase, myozenin-1, the alpha-2 subunit of the Na^+^/K^+^-ATPase, myoglobin, perilipin-4, adenylate kinase isoform AK1, and parvalbumin. The reduction in the adenylate kinase isoform AK1 is an interesting finding and agrees with the first gel-based proteomic profiling of *mdx* hind limb muscles, published in 2003 by Ge *et al.* [38].

**Table 4:** List of changed proteins in 100-day old *mdx* *flexor digitorum brevis* muscle *vs.* age-matched wild type muscle as determined by label-free mass spectrometric analysis.

Accession Number	Protein Name	Tissue Localization	Peptides	Score	ANOVA (p)	Fold Change
P20152	Vimentin	Intermediate filaments	5	256.96	1.85 × 10^−3^	2.39
P07356	Annexin A2	Sarcolemma/basal lamina	3	194.99	1.79 × 10^−3^	2.29
P60710	Actin, cytoplasmic 1	Cytoplasm	2	191.53	7.90 × 10^−3^	2.27
Q00623	Apolipoprotein A-I	Cytoplasm/extracellular space	2	140.56	2.04 × 10^−3^	2.17
P22599	Alpha-1-antitrypsin 1-2	Extracellular region	4	188.28	4.08 × 10^−3^	2.17
P07724	Serum albumin	Extracellular space	2	98.97	2.57 × 10^−3^	2.16
Q921I1	Serotransferrin	Extracellular space	4	216.10	3.21 × 10^−3^	2.01
P11499	Heat shock protein Hsp90-beta (HSPAB1)	Cytoplasm	2	114.79	8.47 × 10^−4^	2.00
P48678	Prelamin-A/C	Nuclear lamina	13	807.87	1.40 × 10^−3^	1.92
P51885	Lumican	Extracellular matrix	3	175.81	6.93 × 10^−3^	1.91
P48036	Annexin A5	Sarcolemma region	2	116.73	1.34 × 10^−2^	1.80
P09103	Protein disulfide-isomerase	Endoplasmic reticulum	2	142.68	5.43 × 10^−4^	1.67
Q02788	Collagen alpha-2(VI) chain	Extracellular matrix	4	246.86	6.62 × 10^−3^	1.62
Q04857	Collagen alpha-1(VI) chain	Extracellular matrix	3	192.60	3.43 × 10^−3^	1.61
P17751	Triosephosphate isomerase	Cytoplasm/glycolytic particle	2	140.70	9.58 × 10^−3^	−1.53
Q9QYG0	Protein NDRG2	Cytoplasm/nucleoplasm	2	168.53	5.98 × 10^−3^	−1.61
Q9WUB3	Glycogen phosphorylase, muscle	Cytoplasm	2	99.23	1.70 × 10^−2^	−1.61
Q9JK37	Myozenin-1	Actin cytoskeleton	2	149.89	8.00 × 10^−3^	−1.68
Q6PIE5	Na^+^/K^+^-ATPase, alpha-2 subunit	Sarcolemma	4	216.92	8.65 × 10^−3^	−1.71
P04247	Myoglobin	Cytoplasm	3	193.88	4.83 × 10^−3^	−1.81
O88492	Perilipin-4	Cytoplasm	2	138.76	5.52 × 10^−4^	−2.04
Q9R0Y5	Adenylate kinase AK1	Cytoplasm	2	137.26	3.07 × 10^−3^	−2.07
P32848	Parvalbumin, alpha	Cytoplasm	2	113.41	8.62 × 10^−3^	−2.69

### 2.6. Proteomic Analysis of mdx Interosseus Muscle

In the INT muscle, label-free mass spectrometric analysis identified a drastic increase in myosin-3 and myosin-4, as well as elevated levels of annexin A2, vimentin, lamin-A/C, aspirin, elongation factor EEF1A1, serotransferrin, collagen alpha-1(VI) chain, decorin, and serpin B6 (Table 5). In contrast, reductions were observed for the alpha-2 subunit of the Na^+^/K^+^-ATPase, the fast SERCA1 isoform of the sarcoplasmic reticulum Ca^2+^-ATPase and myosin-7.

### 2.7. Summary of Protein Classes with a Changed Abundance in mdx Muscles

**Figure 3:**
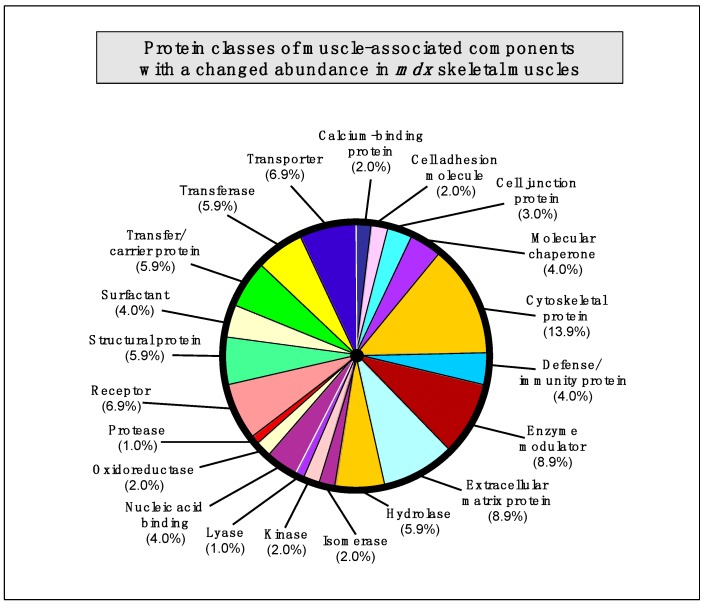
Bioinformatic summary of changed protein classes in *mdx* skeletal muscles, as determined by the software programme PANTHER [40,41]. The graph outlines the clustering of protein classes based on the label-free mass spectrometric analysis of normal *vs.* dystrophic muscles (Table 1, Table 2, Table 3, Table 4, Table 5).

The bioinformatics analysis of altered proteins using the PANTHER database of protein families [40] resulted in the cataloguing of distinct muscle protein categories that had been identified by label-free mass spectrometric analysis in the various *mdx* tissues [41] (Figure 3).

**Table 5:** List of changed proteins in 100-day old *mdx interosseus* muscle *vs.* age-matched wild type muscle as determined by label-free mass spectrometric analysis.

Accession Number	Protein Name	Tissue Localization	Peptides	Score	ANOVA (p)	Fold Change
P13541	Myosin-3 (perinatal MHC)	Myofibrils (myosin complex)	2	299.48	6.78 × 10^−4^	15.70
P13542	Myosin-4 (MHC-IIB)	Myofibrils (myosin complex)	3	502.66	1.99 × 10^−4^	8.88
P07356	Annexin A2	Sarcolemma/basal lamina	2	120.25	6.70 × 10^−5^	2.88
P20152	Vimentin	Intermediate filaments	4	293.05	1.26 × 10^−3^	2.82
P48678	Prelamin-A/C	Nuclear lamina	11	736.17	1.43 × 10^−3^	2.56
Q99MQ4	Asporin	Extracellular matrix	2	93.48	3.76 × 10^−3^	2.53
P10126	Elongation factor 1-alpha 1	Cytoplasm/ribosome	2	87.24	3.21 × 10^−3^	2.27
Q921I1	Serotransferrin	Extracellular space	3	167.69	7.26 × 10^−3^	2.05
Q04857	Collagen alpha-1(VI) chain	Extracellular matrix	2	99.46	2.25 × 10^−2^	1.86
P28654	Decorin	Extracellular matrix	2	162.96	1.49 × 10^−2^	1.79
Q60854	Serpin B6	Cytoplasm	2	113.51	3.31 × 10^−3^	1.66
Q6PIE5	Na^+^/K^+^-ATPase, alpha-2 subunit	Sarcolemma	3	235.21	5.85 × 10^−3^	−1.54
Q8R429	SERCA1 Ca^2+^-ATPase	Sarcoplasmic reticulum	3	163.52	1.49 × 10^−3^	−2.02
Q91Z83	Myosin-7 (cardiac MHC-β)	Myofibrils (myosin complex)	6	528.44	1.84 × 10^−2^	−2.88

The following protein classes exhibited a changed concentration: cytoskeletal protein (13.9%), extracellular matrix protein (8.9%), enzyme modulator (8.9%), enzyme modulator (8.9%), transporter (6.9%), receptor (6.9%), transferase (5.9%), structural protein (5.9%), hydrolase (5.9%), transfer/carrier protein (5.9%), nucleic acid binding protein (4.0%), molecular chaperone (4%), surfactant (4.0%), defense/immunity protein (4.0%), cell junction protein (3.0%), cell adhesion molecule (2.0%), calcium-binding protein (2.0%), cell adhesion molecule (2.0%), oxidoreductase (2.0%), kinase (2%), isomerase (2%), protease (1.0%), and lyase (1.0%).

### 2.8. Identification of Universal Markers of Dystrophinopathy

**Figure 4:**
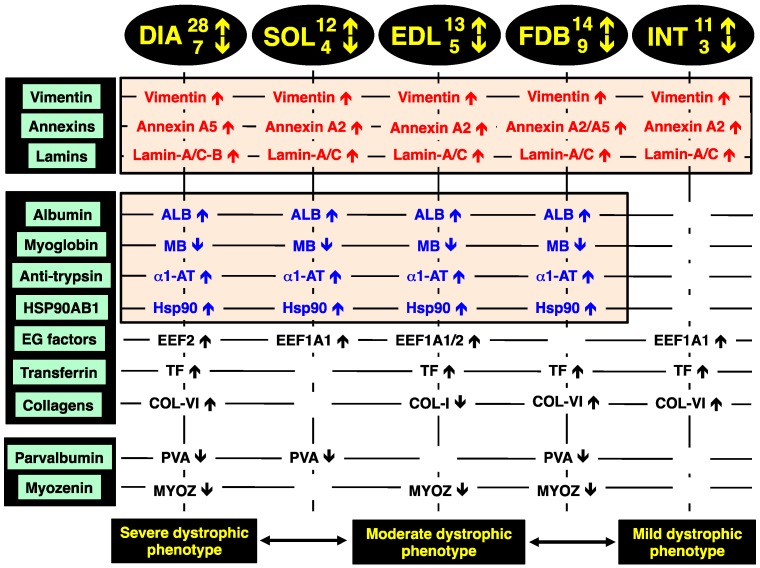
Overview of the universal biomarker signature of dystrophic diaphragm (DIA), *soleus* (SOL), *extensor digitorum longus* (EDL), *flexor digitorum brevis* (FDB), and *interosseus* (INT) muscles from the *mdx* animal model of Duchenne muscular dystrophy, as revealed by label-free mass spectrometry.

The comparison of the proteomic data sets revealed that 3 protein species show similar changes in all dystrophic tissue specimens investigated in this study, *i.e.*, a significant increase in the intermediate filament protein vimentin, the Ca^2+^-dependent membrane binding protein annexin (isoforms A2, A5), and the nuclear envelope protein lamin (isoforms A/C, B). These new universal biomarker candidates are summarised in Figure 4.

In addition, all investigated *mdx* muscles, with the exception of INT preparations, showed analogous changes in four other muscle-associated proteins. Increases were demonstrated for albumin, anti-trypsin protein and the molecular chaperone Hsp90-beta (HSPAB1) and a decrease established for the cytoplasmic oxygen-carrier myoglobin. Additional proteins with a differential expression pattern in some of the analysed dystrophin-deficient muscles were identified as elongation factors EEF1A1 and EEF2, the iron-binding protein transferrin, various isoforms of collagen, the cytosolic Ca^2+^-binding protein parvalbumin, and the Z-line α-actinin binding protein myozenin (Figure 4).

The increase in the intermediate filament protein vimentin is probably a compensatory mechanism to stabilize the weakened cytoskeletal network and to rescue the load-bearing function of the fibre interior that lacks the dystrophin lattice in muscular dystrophy. Although a previous report has shown that vimentin is only transiently expressed during myotube maturation [42], vimentin can act synergistically to desmin and support the structural backbone of intermediate filaments [43]. The finding agrees with previous gel-based proteomic analyses of dystrophic mouse and dog skeletal muscles [23,24,25,32,34,36], as well as a comprehensive *in vivo* SILAC proteomic study of the *mdx* mouse [33] and a label-free mass spectrometric survey of the highly fibrotic *mdx-4cv* diaphragm [35]. This large number of corresponding proteomic results establishes this intermediate filament protein as a reliable and versatile muscle-associated biomarker candidate for evaluating animal models of dystrophinopathy [28].

The drastic increase in annexin isoforms A2 and A5 suggests impaired Ca^2+^-handling and an altered membrane organization in dystrophin-deficient fibres [44]. Since annexins are linked to the maintenance of the extracellular matrix and the actin-associated cytoskeletal network in muscle [45], their apparent up-regulation could also be an adaptive response and attempt to partially substitute for the loss of the dystrophin-actin axis [26]. The dystrophic *grmd* dog model of Duchenne muscular dystrophy also exhibited an increased level of annexins in the *vastus lateralis* muscle [36]. Since lamins are nuclear intermediate filament proteins that provide nuclear stability and support the structural linkage between muscle nuclei and the cytoskeleton [46], increased levels of lamin isoforms A/C and B probably enhance the assembly of lamin-based fibrous structures. This would maintain the inner nuclear membrane structure during degeneration-regeneration cycles and stabilize muscle fibres affected by inflammatory processes.

Altered albumin levels in dystrophic muscles may reflect disturbed oxidative metabolism and/or is connected to an increased permeability of the Dp427-deficient sarcolemma, which represents a major pathological feature in muscular dystrophies [47]. The up-regulation of alpha-1-antitrypin [48], which functions as a protective anti-protease and anti-inflammatory factor [49], might be an adaptation of dystrophic muscle tissues in response to fibre degeneration and inflammation [50]. Anti-trypsin, also known as serpina 1d protein, was also shown to be greatly increased in FDB muscles by a gel-based study [24]. The change in the Serpin B6 serine protease inhibitor was also reported by Ge *et al.* [38]. The loss of the cytoplasmic oxygen-carrier myoglobin, usually present at high levels in oxidative skeletal muscle fibres [51], could be due to leakage from dystrophic muscles and therefore be indicative of progressive disintegration of the dystrophic sarcolemma. Since elongation factors control muscle protein synthesis by delivering the aminoacyl-tRNA to the ribosome and thereby ensuring the proper elongation of the nascent polypeptide chain [52], increased levels of EEF1A1 and EEF2 may be involved in regenerative processes. Disturbed iron metabolism is indicated by the increased concentration of the iron-binding protein transferrin [53,54] in muscular dystrophy.

The drastic increase of collagen in the *mdx* diaphragm agrees with fibrosis-associated changes in dystrophinopathy [55,56,57] and confirms the results from previous analyses of dystrophic muscles [34,35]. The drastic decrease of the cytosolic Ca^2+^-binding protein parvalbumin [58,59] in dystrophic muscle indicates abnormal Ca^2+^-buffering and supports the calcium hypothesis of dystrophinopathy [60,61,62,63,64]. A preferential susceptibility of differing fibre populations may be linked to lowered levels of parvalbumin [24,35,37]. An interesting finding is the increase in the large heat shock protein Hsp90 that represents a major ATP-dependent molecular chaperone [27]. Hsp90 is involved in the activation and stabilization of many signalling proteins involved in cellular pathways. Changes in this molecular chaperone and its cyto-protective action indicate increased levels of cellular stress in muscular dystrophy [65,66] and might be a suitable marker of progressive dystrophic alterations [28]. Changes in the α-actinin binding protein myozenin of the Z-disc region [67] are also a potential new indicator of dystrophinopathy-related abnormalities within the complex arrangement of the contractile apparatus [68].

In order to relate the mass spectrometric analysis presented here with previous studies, significant proteomic hits were compared to major findings from already published reports on proteome-wide alterations in established model systems of dystrophinopathy. Table 6 and Table 7 summarize the list of major biomarker candidates identified in this report by label-free mass spectrometry and correlates the changes in 28 proteins with the proteomic results from 13 previous studies that have focused on the *mdx* mouse, the *mdx-4cv* mouse and the *grmd* dog models of Duchenne muscular dystrophy [21,22,23,24,25,32,33,34,35,36,37,38,39]. The tables outline the identified proteins, whereby certain proteomic hits exhibit dystrophy-related changes in more than one isoform [26,27,28], their subcellular localization and a list of analysed skeletal muscle types and animal models. Importantly, Table 6 illustrates that the observed increase in the intermediate filament component vimentin has also been shown in a considerable number of other studies [21,22,23,24,25,32,33,34,35,36] and Table 7 confirms that the significant decrease in the cytosolic Ca^2+^-binding protein parvalbumin was also identified in previous proteomic investigations [24,33,35,37] of dystrophin-deficient skeletal muscles. This establishes these two muscle-associated proteins with opposite changes in their concentration in dystrophic fibres as excellent analytical tools for the future assessment of animal models of X-linked muscular dystrophy.

**Table 6:** Overview of increased biomarker candidates and correlation to previously published proteomic studies of secondary changes in animal models of dystrophinopathy.

Protein Name	Tissue Localization	Animal Models	Skeletal Muscles	References
Vimentin	Intermediate filaments	*mdx*, *mdx-4cv*, *grmd*	DIA, EDL, FDB, GAS, INT, SOL, VL	[21,23,24,25,32,33,34,35,36]
Annexin A1, A2, A5, A6	Sarcolemma region	*mdx*	DIA, EDL, FDB, INT, SOL	[25,33,37,38]
Lamin A/C, B	Nuclear lamina	*mdx*, *mdx-4cv*	DIA, EDL, FDB, INT, SOL	[35]
Myosin, embryonic MHC-3, MHC-8	Myofibrils	*mdx*, *mdx-4cv*	DIA, GAS, INT	[33,35]
Obscurin	Contractile apparatus	*mdx*, *mdx-4cv*	DIA	[35]
Hemopexin	Extracellular space	*mdx*, *grmd*	DIA, SOL	[36]
Collagens, especially COL-VI	Extracellular matrix	*mdx*, *mdx-4cv*	DIA, FDB, GAS, SOL	[23,24,34,35]
Histone	Nucleus	*mdx*, *mdx-4cv*	DIA, EDL, GAS	[23,25,33,35]
Serum albumin	Extracellular space	*mdx*, *grmd*	DIA, EDL, FDB, GAS, SOL, VL	[23,32,36]
Tubulin	Microtubules	*mdx*, *mdx-4cv*, *grmd*	DIA, GAS, VL	[23,33,35,36]
78 kDa glucose-regulated protein	Endoplasmic reticulum	*mdx*	DIA, GAS	[25,33,37]
Transferrin	Extracellular space	*mdx*, *mdx-4cv*	DIA, EDL, FDB, GAS, INT	[23,33,35,37,39]
Filamin A, C	Actin cytoskeleton	*mdx*, *mdx-4cv*	DIA, GAS	[33,35]
Proteoglycans (aporin, lumican, prolargin, biglycan, decorin)		*mdx*, *mdx-4cv*	DIA, INT	[35,37]
Elongation factors	Cytoplasm/ribosome	*mdx*	DIA, EDL, GAS, INT, SOL	[23]
Heat shock protein Hsp90	Cytoplasm	*mdx*, *mdx-4cv*	DIA, EDL, FDB, GAS, SOL	[23,35]
Heat shock protein Hsp70	Cytoplasm	*mdx*	DIA, GAS	[23,37,39]
Small heat shock proteins (HspB1, HspB5, HspB7)	Cytoplasm	*mdx*	DIA, GAS, INT, SOL	[21,23,24]
Desmin	Intermediate filaments	*mdx*, *mdx-4cv*	DIA, EOM, GAS	[32,33,35]
Vinculin	Cytoskeleton	*mdx*, *mdx-4cv*	DIA, GAS	[33,35]
Anti-trypsin	Extracellular region	*mdx*	DIA, EDL, FDB, GAS, SOL	[33,38]

Abbreviations used: DIA, diaphragm; EDL, *extensor digitorum longus*; EOM, extraocular muscle; FDB, *flexor digitorum brevis*; GAS, *gastrocnemius*; INT, *interosseus*; SOL, *soleus*; VL, *vastus lateralis*.

**Table 7:** Overview of decreased biomarker candidates and correlation to previously published proteomic studies of secondary changes in animal models of dystrophinopathy.

Protein Name	Tissue Localization	Animal Models	Skeletal Muscles	References
Parvalbumin	Cytoplasm	*mdx*, *mdx-4cv*	DIA, FDB, GAS, INT, SOL	[24,33,35,37]
Myoglobin	Cytoplasm	*mdx*	DIA, EDL, FDB, SOL	[24,37]
Fatty acid-binding protein FABP3	Cytoplasm	*mdx*	DIA, SOL, GAS	[33,37]
Carbonic anhydrase CA3	Cytoplasm	*mdx*	DIA	[21]
Perilipin	Cytoplasm	*mdx*, *mdx-4cv*	DIA, FDB	[35]
Myozenin	Actin cytoskeleton	*mdx*, *mdx-4cv*	DIA, EDL, FDB, GAS	[34,35]
Glycogen phosphorylase, muscle	Cytoplasm	*mdx*, *mdx-4cv*	DIA, EDL, GAS	[23,24,35]

Abbreviations used: DIA, diaphragm; EDL, *extensor digitorum longus*; FDB, *flexor digitorum brevis*; GAS, gastrocnemius; INT, *interosseus*; SOL, *soleus*.

From the comparative analysis of the existing literature, in conjunction with the proteomic findings from this report, a variety of other promising biomarker candidates other than vimentin and parvalbumin have been identified. Increased proteins included various annexins, desmin, vinculin, tubulin, lamin, embryonic isoforms of myosin heavy chains, obscurin, hemopexin, various histones, albumin, transferrin, filamin, elongation factors, a variety of molecular chaperones and anti-trypsin, as well as collagen and associated proteoglycans (Table 6). Decreased proteins with a great potential to be useful for studying particular aspects of the molecular pathogenesis of dystrophinopathy or for the systematic monitoring of experimental therapies are myoglobin, the fatty acid-binding protein isoform FABP3, carbonic anhydrase CA3, perilipin, myozenin, and the muscle isoform of glycogen phosphorylase (Table 7). Since the identified proteins cover a wide range of cellular and structural activities in skeletal muscle tissues, their combined usage for the determination of changes in a biomarker signature would cover the maintenance of the cytoskeletal network, the extracellular matrix, energy metabolism, metabolite transportation, the cellular stress response, the excitation-contraction-relaxation cycle and other core activities.

### 2.9. Immunoblotting Survey of mdx Skeletal Muscles

**Figure 5:**
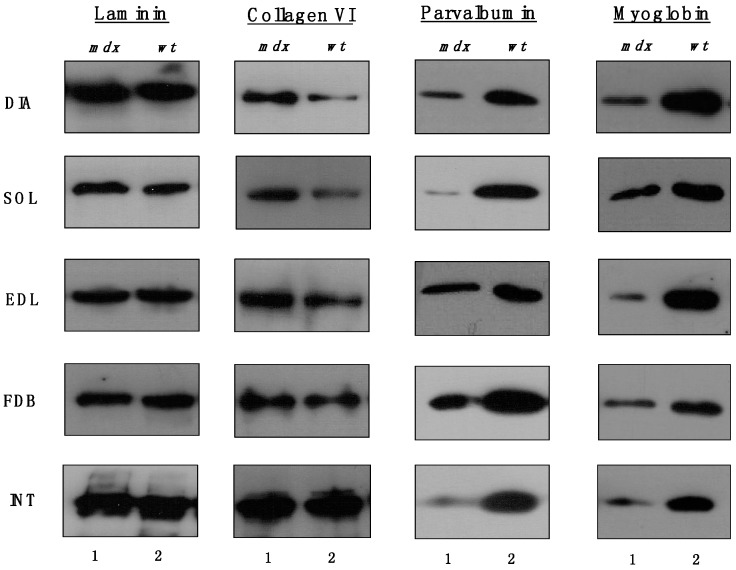
Comparative immunoblotting survey of dystrophic diaphragm (DIA), *soleus* (SOL), *extensor digitorum longus* (EDL), *flexor digitorum brevis* (FDB), and *interosseus* (INT) muscles from the *mdx* (lane 1) animal model of Duchenne muscular dystrophy *vs.* wild type (*wt*; lane 2) muscles. Antibody labelling was used to determine the concentration of laminin, collagen, parvalbumin and myoglobin.

The immunoblots shown in Figure 5 confirmed the relatively unchanged concentration levels of laminin in dystrophin-deficient skeletal muscles and the tendency of increased collagen levels in all *mdx* preparations except INT muscle. All *mdx* muscles exhibited drastic decreases in myoglobin and parvalbumin, which agrees with previous findings [26,27,28].

**Figure 6:**
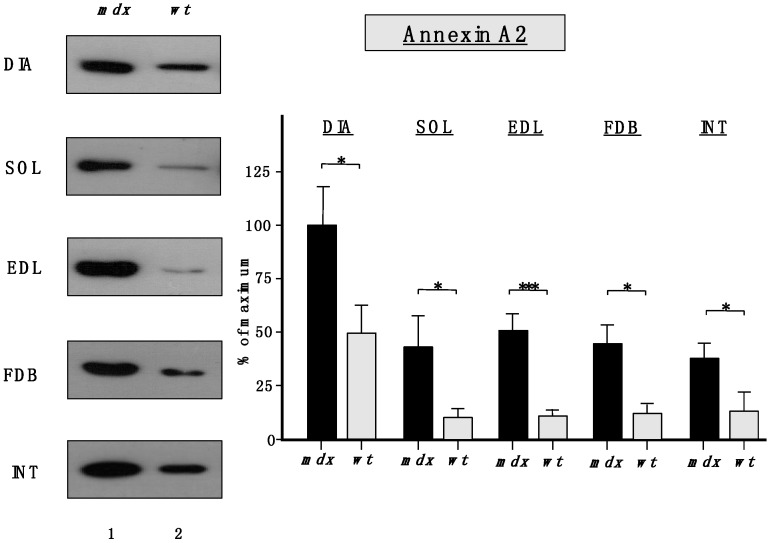
Comparative immunoblot analysis of dystrophic diaphragm (DIA), *soleus* (SOL), *extensor digitorum longus* (EDL), *flexor digitorum brevis* (FDB), and *interosseus* (INT) muscles from the *mdx* (lane 1) animal model of Duchenne muscular dystrophy *vs.* wild type (*wt*; lane 2) muscles. Antibody labeling was used to determine the concentration of annexin isoform A2 and is shown on the left side of this figure. The graphical representation of the immuno-decoration levels for annexin in *mdx vs.* wild type (*wt*) muscles is shown on the right side of this figure (Student’s *t*-test, unpaired; *n* = 4; * *p* < 0.05; *** *p* < 0.001).

Importantly, Figure 6 clearly confirmed the findings from the proteomic survey of *mdx* muscles and demonstrated that annexins are significantly increased in the *mdx* muscles investigated in this study. The immunoblot analysis focused on annexin isoform A2.

**Figure 7:**
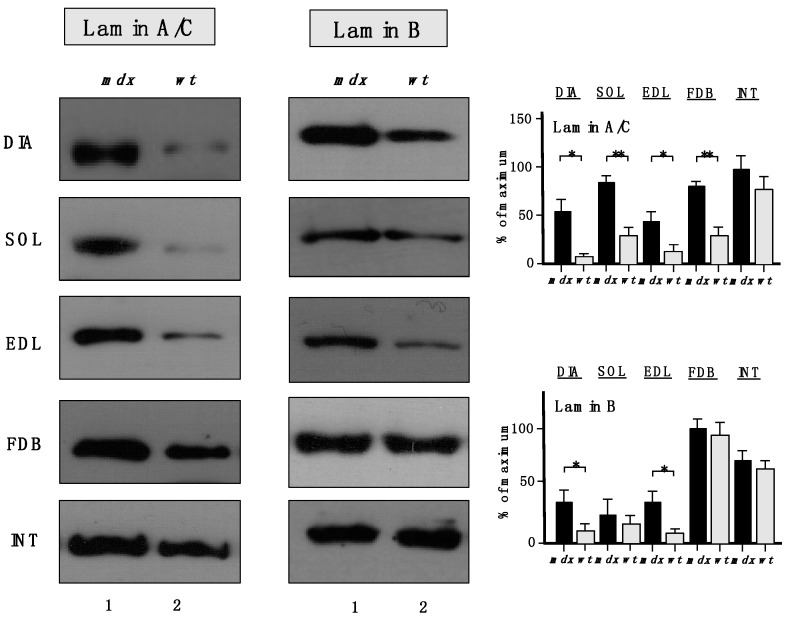
Comparative immunoblot analysis of dystrophic diaphragm (DIA), *soleus* (SOL), *extensor digitorum longus* (EDL), *flexor digitorum brevis* (FDB), and *interosseus* (INT) muscles from the *mdx* (lane 1) animal model of Duchenne muscular dystrophy *vs.* wild type (*wt*; lane 2) muscles. Antibody labelling was used to determine the concentration of lamin isoform A/C and lamin isoform B, as shown on the left side of the figure. Graphical representation of the immuno-decoration levels for lamin A/C and lamin B in *mdx vs.* wild type (*wt*) muscles is shown on the right side of the figure (Student’s *t*-test, unpaired; *n* = 4; * *p* < 0.05; ** *p* < 0.01).

The increased levels of the nuclear envelope lamins, as suggested by proteomic analyses, were confirmed to be significant for lamin-A/C, with the exception of INT muscle (Figure 7). A drastic increase of lamin-B levels was also shown to occur in DIA and EDL muscles.

**Figure 8:**
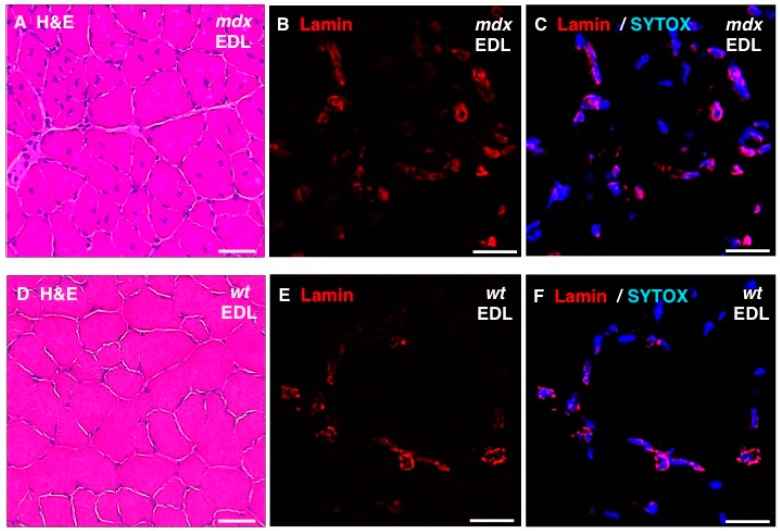
Histochemical analysis and immunofluorescence labelling of lamin in dystrophic *mdx vs.* wild type (*wt*) skeletal muscles. Shown are transverse sections of *extensor digitorum longus* (EDL) muscles from 100-day old *mdx* (**A**–**C**) and wild type (**D**–**F**) mice. Cryosections were stained with hematoxylin and eosin (H & E) (**A**,**D**) or analysed by immunofluorescence microscopy using antibodies to lamin A/C (**B**,**C**,**E**,**F**). In panels (**C**,**F**), merged images of SYTOX-based nucleic acid staining of nuclei (blue) and immunofluorescence labelling of lamin (red) are shown. In panels (**A**,**D**): bar equals 50μm; and in panels (**B**,**C**,**E**,**F**): bar equals 20 μm.

In addition, standard histochemical staining of transverse tissue sections with hematoxylin and eosin was used to estimate changes in the position and number of nuclei in dystrophic EDL muscles (Figure 8A,D). Immunofluorescence microscopy was employed to illustrate the localization and amounts of lamin in *mdx* fibres (Figure 8B,E) and these findings were correlated to the position of nuclei as judged by labelling of nucleic acids with SYTOX (Figure 8C,F). The histochemical analysis suggests that *mdx* fibres contain a considerably higher degree of central nucleation and an overall increased number of nuclei, which agrees with the previous histological profiling of the *mdx* mouse model of Duchenne muscular dystrophy [24,69]. Immunofluorescence microscopy revealed specific labelling of the nuclear envelope by antibodies to lamin A/C and the comparison between *mdx* and wild type EDL muscles suggests increased levels of lamin in dystrophin-deficient muscles.

**Figure 9:**
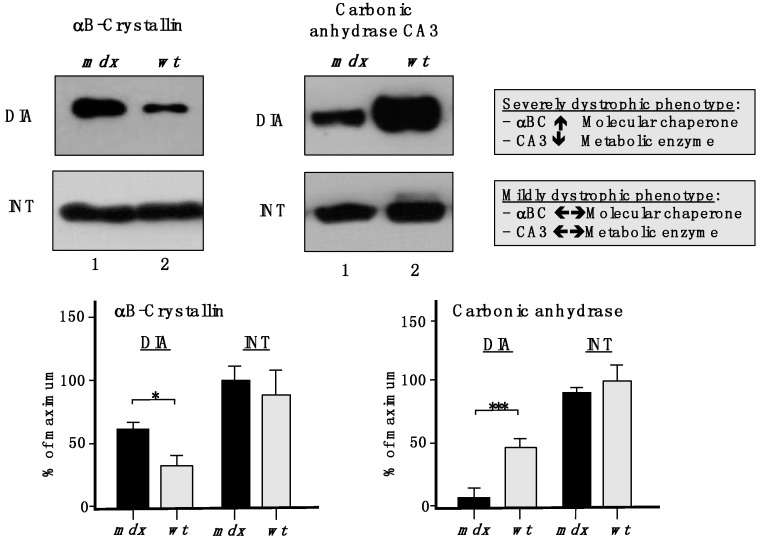
Comparative immunoblotting to illustrate the drastic differences in biomarker concentrations between a severely dystrophic phenotype, the *mdx* diaphragm (DIA), *vs.* a mildly affected muscle, the *mdx interosseus* (INT) muscle. Lane 1 and 2 represent specimens from the *mdx* animal model of Duchenne muscular dystrophy *vs.* wild type (*wt*) specimens, respectively. Antibody labelling was used to determine the concentration of the small heat shock protein αB-crystallin and carbonic anhydrase isoform CA3, as shown in the upper part of the figure. The graphical representation of the immuno-decoration levels for αB-crystallin and carbonic anhydrase in *mdx vs.* *wt* muscles is shown in the lower part of the figure (Student’s *t*-test, unpaired; *n* = 4; * *p* < 0.05; *** *p* < 0.001).

To show the drastic differences between a severely dystrophic phenotype *vs.* a mildly affected muscle, immunoblotting with two marker proteins is shown in Figure 9. In contrast to a drastic increase of the small heat shock protein αB-crystallin and a decrease in the CA3 isoform of carbonic anhydrase in *mdx* DIA muscle, both proteins exhibited comparable levels in *mdx* INT muscle *vs.* wild type INT muscle.

The findings from the comparative proteomic profiling and the select immunoblotting survey of the *mdx* diaphragm, *interosseus*, *flexor digitorum brevis*, *soleus*, and *extensor digitorum longus* muscles clearly reflect differences in the number and extent of expression changes in proteins in severely *vs.* moderately or mildly dystrophic skeletal muscles. These pathobiochemical differences are probably due to variations in compensatory or adaptive mechanisms. The different subtypes of skeletal muscles studied in this report exhibit considerable dissimilarities in their contractile properties, their cellular size, motor unit organization, physiological adaptability, fibre type distribution, the extent of their calcium extrusion systems, vulnerability to proteolytic degradation, susceptibility to fibre degeneration, exposure to fatty tissue substitution, and predisposition to progressive myofibrosis. This would explain the differences in proteome-wide alterations related to proteins involved in the excitation-contraction-relaxation cycle, metabolite transportation, glycolysis, oxidative metabolism, the cytoskeletal network, the matrisome and the cellular stress response. Secondary changes in severely affected muscles that lack dystrophin are related to an (i) up-regulation of molecular chaperones, (ii) the compensatory increase in proteins of the intermediate filaments, (iii) the fibrosis-related increase in collagen levels, and (iv) the pathophysiological decrease in calcium binding proteins.

The new set of universal and muscle-associated biomarkers of progressive muscular dystrophy, such as annexin, lamin, and vimentin, can now be employed to establish improved predictive, diagnostic, prognostic and therapy-monitoring approaches focusing on murine models of dystrophinopathy [70]. Besides pharmacological treatments of general muscle wasting and cardio-respiratory complications, using glucocorticoids, diuretics and beta-blockers [71,72,73,74], new efforts to address the progressive nature of X-linked muscular dystrophy lie in cell-based, gene transfer, stop-codon read-through and exon-skipping procedures [75,76,77,78,79,80,81]. Proteomic markers can be extremely helpful in judging the overall effectiveness of these new therapeutic methods, since changes in these muscle proteins can give excellent indications of the reversal of specific damage pathways involved in progressive dystrophinopathies of model organisms [82].

## 3. Experimental Section

### 3.1. Materials

For the comparative proteomic analysis of mildly *vs.* severely affected *mdx* mouse skeletal muscles, materials and analytical grade chemicals were purchased from BioRad Laboratories (Hemel-Hempstead, Hertfordshire, UK) and Amersham Biosciences/GE Healthcare (Little Chalfont, Buckinghamshire, UK). Sequencing grade modified proteases (Lys-C and trypsin) were from Promega (Madison, WI, USA). Protease inhibitor cocktail tablets and chemiluminescence ECL kits were obtained from Roche (Mannheim, Germany). For immunoblotting, Whatman NC transfer membranes were purchased from Invitrogen (Carlsbad, CA, USA). Primary antibodies for immunoblotting were obtained from Santa Cruz Biotechnology, CA, USA (Sc-33701 to β-dystroglycan), Sigma Chemical Company, Dorset, UK (L-9393 to laminin), and Abcam, Cambridge, UK (ab6588 to collagen VI; ab85366 to carbonic anhydrase isoform CA3; ab11427 to parvalbumin; ab13496 to αB-crystallin; ab8984 to lamin-A/C; ab16048 to lamin B; ab41803 to annexin A2; and ab77232 to myoglobin). For immunofluorescence microscopy, a polyclonal rabbit antibody to lamin A/C (H-110) was purchased from Santa Cruz Biotechnology (Santa Cruz, TX, USA). Peroxidase-conjugated secondary antibodies were from Chemicon International (Temecula, CA, USA). The nucleic acid stain SYTOX Green and goat anti-rabbit IgG Alexa Fluor 647 conjugate was obtained from Invitrogen (Darmstadt, Germany). Tissue-Tek O.C.T. compound was from Sakura (Alphen aan de Rijn, Netherlands) and the mounting media Neo-Mount and Mowiol were purchased from Merck-Millipore (Schwalbach, Germany). All other chemicals were obtained from Sigma Chemical Company (Dorset, UK).

### 3.2. Preparation of Protein Extracts from the mdx Mouse Model of Duchenne Muscular Dystrophy

In order to compare the proteomic profile of dystrophin-deficient skeletal muscles with a highly degenerative *vs.* a mildly to moderately dystrophic phenotype, diaphragm, *interosseus*, *flexor digitorum brevis*, *soleus*, and *extensor digitorum longus* muscle were prepared from 100-day old male *mdx* mice, using a previously optimized method [24]. Wild type controls and *mdx* mice were obtained from the Animal Facility of the University Medicine Greifswald, Germany. Mice were kept under standard conditions and all procedures were carried out in accordance with German and Irish guidelines on the use of animals for scientific experiments; approved by the District Veterinary Office in Anklam, Germany. Animals were sacrificed by cervical dislocation after short ether anaesthesia and individual subtypes of skeletal muscles dissected and immediately quick-frozen in liquid nitrogen. For the comparative proteomic analysis of *mdx* tissues, muscle samples were transported to Maynooth University on dry ice and stored at −80 °C prior to usage. Tissue specimens were homogenised in a lysis buffer containing 7 M urea, 2 M thiourea, 65 mM CHAPS, 100 mM DTT; at a ratio of 1:10 (*w*/*v*). In order to prevent the potential proteolytic degradation of sensitive muscle proteins and aid the extraction process, the homogenization buffer was supplemented with a protease inhibitor cocktail and DNAase, respectively, as previously described in detail [83]. Muscle preparations were homogenised with a hand-held homogenizer model IKA T10 Basic Homogenizer from Fisher Scientific (Dublin, Ireland). Crude muscle extracts were then incubated for 2.5 h at 4 °C with gentle agitation using a Thermomixer from Eppendorf (Hamburg, Germany). Samples were centrifuged at 4 °C for 20 min at 14,000 *g* and the protein-containing supernatant fraction from both normal and *mdx* preparations were then carefully removed and used for the comparative proteomic analysis using label-free mass spectrometry. Protein concentrations were determined by the Bradford assay protocol [84].

### 3.3. Label-Free LC-MS/MS Analysis

Building on the previous gel-based analysis of various skeletal muscle subtypes [24], samples were separated here by a liquid chromatographic method. The proteomic profiling of 5 different subtypes of skeletal muscles from mutant *mdx vs.* wild type mice was carried out with a total of 40 different specimens. This included 4 biological repeats each of the 5 muscle subtypes (DIA, EDL, SOL, FDB, INT) from wild type mice and 4 biological repeats each of the 5 muscle subtypes (DIA, EDL, SOL, FDB, INT) from *mdx* mice. Preparation of individual protein fractions for label-free LC-MS/MS analysis was carried out in accordance with a previously optimised method [83]. Crude skeletal muscle protein samples were pre-treated with the ReadyPrep 2D clean up kit from BioRad Laboratories (Hemel-Hempstead, Hertfordshire, UK). The protein pellets created from the clean up kit were re-suspended in a label-free solubilisation buffer containing 6 M urea, 2 M thiourea and 10 mM Tris-Cl, pH 8.0 in LC-MS grade water. Re-suspended protein samples were then carefully vortexed, sonicated and centrifuged to ensure pellets were fully re-suspended. For label-free MS analysis, volumes were initially equalised with label-free solubilisation buffer and kept to a minimum. All samples were reduced for 30 min with 10 mM DTT and alkylated for 20 min in the dark with 25 mM iodoacetamide in 50 mM ammonium bicarbonate. Proteolytic digestion was initially carried out with Lys-C at a ratio of 1:100 (protease/protein) for 4 h at 37 °C. Samples were then diluted with 50 mM ammonium bicarbonate four times that of the initial volume. The second digestion step using trypsin was performed at a ration of 1:25 (protease/protein) overnight at 37 °C. Ahead of MS analysis the digested protein preparations were finally diluted at a ratio of 3:1 (*v*/*v*) with 2% TFA in 20% ACN, and then again vortexed and sonicated to ensure an even protein suspension.

An Ultimate 3000 nanoLC system (Dionex) coupled to a an LTQ Orbitrap XL mass spectrometer from Thermo Fisher Scientific (Dublin, Ireland) was used for the nano LC-MS/MS analysis of muscle proteins in the Proteomics Facility of the National Institute for Cellular Biotechnology, Dublin City University, as previously described [85,86]. Digested peptide mixtures (5 μL volume) were loaded onto a C18 trap column (C18 PepMap, 300 μm id × 5 mm, 5 μm particle size, 100 Å pore size; Dionex). Desalting was carried out at a flow rate of 25 μL/min in 0.1% TFA and 2% ACN for 5 min. The trap column was switched on-line with an analytical PepMap C18 column (75 μm id × 500 mm, 3 μm particle, and 100 Å pore size; Dionex). Peptides generated from skeletal muscle proteins were eluted with the following binary gradients: solvent A (2% ACN and 0.1% formic acid in LC-MS grade water) and 0%–25% solvent B (80% ACN and 0.08% formic acid in LC-MS grade water) for 240 min and 25%–50% solvent B for a further 60 min. The column flow rate was set to 350 nL/min. Data was acquired with Xcalibur software, version 2.0.7 (Thermo Fisher Scientific). The MS apparatus was operated in data-dependent mode and externally calibrated. Survey MS scans were acquired in the Orbitrap in the 400–1800 *m*/*z* range with the resolution set to a value of 30,000 at *m*/*z* 400 and lock mass set to 445.120025 u. CID fragmentation was carried out in the linear ion trap with up to three of the most intense ions (1+, 2+ and 3+) per scan [86]. Within 40 s, a dynamic exclusion window was applied. A normalised collision energy of 35%, an isolation window of 3 *m*/*z*, and one microscan were used to collect suitable tandem mass spectra [87].

### 3.4. Quantitative Profiling by Label-Free LC-MS/MS Analysis

Progenesis label-free LC-MS software version 3.1 from Non-Linear Dynamics (Newcastle upon Tyne, UK) was used to process the raw data generated from LC-MS/MS analysis. Data alignment was based on the LC retention time of each sample, allowing for any drift in retention time given and adjusted retention time for all runs in the analysis [86]. A reference run was established with the sample run that yielded most features (*i.e.*, peptide ions). The retention times of all of the other runs were aligned to this reference run and peak intensities were then normalized. Prior to exporting the MS/MS output files to MASCOT (www.matrixscience.com) for protein identification, a number of criteria were employed to filter the data. This data included (i) peptide features with ANOVA < 0.05 between experimental groups, (ii) mass peaks (features) with charge states of +1, +2 and +3, and (iii) greater than one isotope per peptide [83]. A MASCOT generic file was generated from all exported MS/MS spectra from Progenesis software. The MASCOT generic file was used for peptide identification with MASCOT (version 2.2) and searched against the UniProtKB-SwissProt database (downloaded in January 2013) with 16,638 proteins (taxonomy: *Mus musculus).* The following search parameters were used for protein identification: (i) MS/MS mass tolerance set at 0.5 Da; (ii) peptide mass tolerance set to 20 ppm; (iii) carbamidomethylation set as a fixed modification; (iv) up to two missed cleavages were allowed; and (v) methionine oxidation set as a variable modification. On average, 3 out of 4 peptides were identified without a missed cleavage. For further consideration and re-importation back into Progenesis LC-MS software for further analysis, only peptides with ion scores of 40 and above were chosen. Importantly, the following criteria were applied to assign a muscle-associated protein as differentially expressed: (i) an ANOVA score between experimental groups of ≤ 0.05, (ii) proteins with ≥ 2 peptides matched, and (iii) a MASCOT score > 40 [85].

The bioinformatics analysis of potential protein interactions was carried out with standard software programmes and applied to catalogue the clustering of molecular functions and to identify potential protein interactions of the MS-identified muscle proteins with a changed concentration in the dystrophic *mdx* skeletal muscles [40]. Analyses were performed with the PANTHER (http://pantherdb.org; version 8.1) comprehensive database of protein families for the cataloguing of molecular functions [41].

### 3.5. Verification of Proteomic Findings Using Comparative Immunoblotting

In order to verify potential concentration changes of specific proteins in highly degenerative *vs.* mildly to moderately affected *mdx* muscles and confirm the proteomic data of this study, diaphragm, *interosseus*, *flexor digitorum brevis*, *soleus*, and *extensor digitorum longus* muscle were analysed by comparative immunoblotting surveys. Gel electrophoretic separation, membrane transfer and antibody incubation were performed by previously optimized methods [88]. The one-dimensional separation of muscle proteins from dystrophic *vs.* normal mice was performed using standard 10% PAGE gels. The electrophoretic transfer to Whatman Protan nitrocellulose sheets was carried out using a standardized wet transfer technique at 100 V for 70 min and 4 °C in a Transblot Cell from BioRad Laboratories (Hemel-Hempstead, Hertfordshire, UK). Membrane sheets were blocked for 1 h in a protein solution (5% (*w*/*v*) fat-free milk powder in phosphate-buffered saline) to prevent non-specific antibody binding. Nitrocellulose sheets were incubated in appropriately diluted primary antibody for a minimum of 3 h at 4 °C with gentle agitation. Membranes were thoroughly washed and incubated with peroxidase-conjugated secondary antibodies for 1 h at room temperature with gentle agitation. Membranes were washed again and antibody-labelled protein bands were visualised by the enhanced chemiluminescence method. Densitometric scanning and analysis of immunoblots was performed with a HP PSC-2355 scanner and ImageJ (NIH, USA) and Graph-Pad Prism software (San Diego, CA, USA).

### 3.6. Histochemical Analysis and Immunofluorescence Microscopy

Standard histochemical analysis and optimized immunofluorescence microscopy was carried out to characterize select *mdx* muscle specimens used in this study. Freshly dissected EDL muscles from 100-day old *mdx* and age-matched wild type mice were embedded in Tissue-Tek O.C.T. compound and frozen in −90 °C tempered petroleum ether [89]. Transverse sections of 10 µm thickness were cut at −25 °C using a Frigocut 2800n cryostat (Leica, Wetzlar, Germany) and air-dried overnight. For a histological overview, tissue sections were stained with hematoxylin and eosin (H & E) according to standard protocols [24], dehydrated in alcohol and mounted with Neo-Mount (Merck). For immunolabelling of lamin, acetone-fixed sections were initially blocked in a solution of phosphate-buffered saline (pH 7.4) containing 10% (*v*/*v*) normal goat serum and 0.5% (*v*/*v*) saponin and then incubated with a polyclonal rabbit antibody against lamin A/C. Primary antibodies were detected by a Alexa Fluor 647 conjugate and nuclei were counter-stained with the high-affinity nucleic acid stain SYTOX Green (Invitrogen). Samples were mounted with Mowiol (Merck-Millipore). All images were taken with an inverse fluorescence microscope model BZ9000 from Keyence Corporation (Mechelen, Belgium).

## 4. Conclusions

Label-free mass spectrometry, in combination with comparative immunoblotting, was successfully employed for the systematic identification of proteome-wide changes in mildly affected muscles *vs.* severely degenerated muscles from the *mdx* mouse model of dystrophinopathy. Striking intra-muscular increases in annexin, vimentin, and lamin were identified that might be exploitable for the future establishment of a comprehensive diagnostic biomarker signature in murine studies. Compensatory mechanisms and/or cellular variances may be responsible for differing secondary changes in individual *mdx* muscles. Differential markers were shown to be albumin, anti-trypsin, myoglobin, Hsp90, elongation factors, transferrin, collagens, parvalbumin, and myozenin. These proteins can now be further characterized to determine their potential usefulness as diagnostic, prognostic or therapy-monitoring markers in the field of animal model research and its biomedical applications.
